# Genomic and genetic analyses of diversity and plant interactions of *Pseudomonas fluorescens*

**DOI:** 10.1186/gb-2009-10-5-r51

**Published:** 2009-05-11

**Authors:** Mark W Silby, Ana M Cerdeño-Tárraga, Georgios S Vernikos, Stephen R Giddens, Robert W Jackson, Gail M Preston, Xue-Xian Zhang, Christina D Moon, Stefanie M Gehrig, Scott AC Godfrey, Christopher G Knight, Jacob G Malone, Zena Robinson, Andrew J Spiers, Simon Harris, Gregory L Challis, Alice M Yaxley, David Harris, Kathy Seeger, Lee Murphy, Simon Rutter, Rob Squares, Michael A Quail, Elizabeth Saunders, Konstantinos Mavromatis, Thomas S Brettin, Stephen D Bentley, Joanne Hothersall, Elton Stephens, Christopher M Thomas, Julian Parkhill, Stuart B Levy, Paul B Rainey, Nicholas R Thomson

**Affiliations:** 1 Centre for Adaptation Genetics and Drug Resistance and Department of Molecular Biology and Microbiology, Tufts University School of Medicine, 136 Harrison Avenue, Boston, MA 02111, USA; 2 Pathogen Genomics, The Wellcome Trust Sanger Institute, Wellcome Trust Genome Campus, Hinxton, Cambridge, CB10 1SA, UK; 3 Department of Plant Sciences, University of Oxford, South Parks Road, Oxford OX1 3RB, UK; 4 School of Biological Sciences, The University of Reading, Whiteknights, Reading RG6 6AJ, UK; 5 New Zealand Institute for Advanced Study, Massey University, Private Bag 102 904, North Shore Mail Centre, Auckland, New Zealand; 6 Department of Chemistry, University of Warwick, Gibbet Hill Road, Coventry CV4 7AL, UK; 7 Department of Biological Sciences, University of Warwick, Gibbet Hill Road, Coventry CV4 7AL, UK; 8 DOE Joint Genome Institute, Bioscience Division, Los Alamos National Laboratory, Los Alamos, NM 87545, USA; 9 Genome Biology Program, Department of Energy's Joint Genome Institute, 2800 Mitchell Drive, Walnut Creek, CA 94598, USA; 10 Department of Biosciences, The University of Birmingham, Edgbaston, Birmingham B15 2TT, UK; 11 Allan Wilson Centre for Molecular Ecology and Evolution, Massey University Auckland, Private Bag 102 904, North Shore Mail Centre, Auckland, New Zealand; 12 Current address: AgResearch Limited, Grasslands Research Centre, Private Bag 11008, Palmerston North, New Zealand; 13 Current address: School of Life Sciences, University of the West of England, Bristol, Frenchay Campus, Coldharbour Lane, Bristol BS16 1QY, UK; 14 Current address: Faculty of Life Sciences, The University of Manchester, Oxford Road, Manchester M13 9PT, UK; 15 Current address: Biozentrum, University of Basel, Klingelbergstrasse 50-70, 4056 Basel, Switzerland; 16 Current address: SIMBIOS Centre, Level 5, Kydd Building, University of Abertay Dundee, Bell Street, Dundee DD1 1HG, UK

## Abstract

**Background:**

*Pseudomonas fluorescens *are common soil bacteria that can improve plant health through nutrient cycling, pathogen antagonism and induction of plant defenses. The genome sequences of strains SBW25 and Pf0-1 were determined and compared to each other and with *P. fluorescens *Pf-5. A functional genomic *in vivo *expression technology (IVET) screen provided insight into genes used by *P. fluorescens *in its natural environment and an improved understanding of the ecological significance of diversity within this species.

**Results:**

Comparisons of three *P. fluorescens *genomes (SBW25, Pf0-1, Pf-5) revealed considerable divergence: 61% of genes are shared, the majority located near the replication origin. Phylogenetic and average amino acid identity analyses showed a low overall relationship. A functional screen of SBW25 defined 125 plant-induced genes including a range of functions specific to the plant environment. Orthologues of 83 of these exist in Pf0-1 and Pf-5, with 73 shared by both strains. The *P. fluorescens *genomes carry numerous complex repetitive DNA sequences, some resembling Miniature Inverted-repeat Transposable Elements (MITEs). In SBW25, repeat density and distribution revealed 'repeat deserts' lacking repeats, covering approximately 40% of the genome.

**Conclusions:**

*P. fluorescens *genomes are highly diverse. Strain-specific regions around the replication terminus suggest genome compartmentalization. The genomic heterogeneity among the three strains is reminiscent of a species complex rather than a single species. That 42% of plant-inducible genes were not shared by all strains reinforces this conclusion and shows that ecological success requires specialized and core functions. The diversity also indicates the significant size of genetic information within the *Pseudomonas *pan genome.

## Abstract 2

Comparison of the genome sequences of three Pseudomonas fluorescens strains reveals a heterogeneity reminiscent of a species complex rather than a single species

## Background

*Pseudomonas fluorescens *is a physiologically diverse species of opportunistic bacteria (gamma-proteobacteria) found throughout terrestrial habitats. The species contributes greatly to the turnover of organic matter and, while present in soil, is abundant on the surfaces of plant roots and leaves. Of the plant-colonizing strains, some, such as isolates SBW25 and Pf-5, positively affect plant health and nutrition [1-3]. The mechanistic bases of these effects remain unclear, but are known to include the production of plant growth hormones, the suppression of pathogens (especially fungi and oomycetes) detrimental to plant health via competitive and/or allelopathic effects, and the direct elicitation of plant defense responses [4].

It has been argued that exploitation of these plant growth promoting bacteria in agriculture requires an improved understanding of the determinants of ecological performance, particularly persistence [5]. To this end, *in vivo *expression technology (IVET) promoter trapping strategies were devised and implemented to identify plant-induced and soil-induced genes [5-9]. In these early studies a number of coding sequences (CDSs) of ecological relevance were found to be up-regulated, including a type III secretion system [10,11], a cellulose biosynthetic locus [6] and a number of CDSs involved in metabolism and protective responses [12-17]. However, the ability to comprehensively identify ecologically important sequences was limited in these previous studies by the use of incomplete genome libraries and the lack of whole genome sequences.

The genome sequence of a single isolate of *P. fluorescens*, Pf-5, has been reported [18]. Although a large number of genes involved in nutrient uptake/degradation and biocontrol were identified in Pf-5, the true diversity within this species was not revealed. To address this issue and to enhance our understanding of the functional ecology of *P. fluorescens*, we have determined the complete nucleotide sequences of two strains from different environmental origins.

SBW25 was isolated in 1989 from the leaf surface of a sugar beet plant grown at the University Farm, Wytham, Oxford, UK [19]. In addition to its use in the study of microbe-plant-soil interactions, SBW25 has become an important model organism for studies on evolutionary processes (for example, [20,21]). Pf0-1 was isolated in 1987 from loam soil in Sherborn, Massachusetts, USA [22].

Here we report the genome sequences of SBW25 and Pf0-1 and the results of a comparative analysis of *P. fluorescens *that includes isolate Pf-5. Our data reveal hitherto unrecognized diversity [23], with the three strains sharing only 61.4% of genes. We also identify highly abundant families of repetitive DNA sequences and describe more than 100 genes that show elevated levels of expression in the plant environment. These plant-induced genes provide a snapshot of how *P. fluorescens *perceives and responds to the plant environment and reveals conservation of strategies among strains for the enhancement of ecological performance.

## Results and discussion

### *P. fluorescens *SBW25 and Pf0-1 genome architecture

The general features of the genomes of *P. fluorescens *SBW25 (6,722,539 bp) and Pf0-1 (6,438,405 bp) are summarized in Table 1. SBW25 is predicted to encode 6,009 CDSs, with a coding density of 88.3%. The genome of Pf0-1 has 5,741 CDSs with a coding density of 90%. These findings compare to 6,144 CDSs predicted for Pf-5 (7,074,893 bp and 88.7% coding density) [18].

**Table 1:** General characteristics of the genomes of *P. fluorescens *strains SBW25, Pf0-1 and Pf-5

*P. fluorescens *strain	SBW25	Pf0-1	Pf-5*
Number of bases	6,722,539 bp	6,438,405 bp	7,074,893 bp
Number of CDSs	6,009	5,741	6,144
Pseudogenes	88	9	NA
Coding percentage	88.3%	90%	88.7%
%GC	60.5	60.62	63.3
tRNAs	66	73	71
rRNA genes (clusters)	16 (5)	19 (6)	15 (5)
Intergenic repeat families	6 (with a total of 1,199 repeats)	9 (with a total of 231 repeats)	5 (with a total of 748 repeats)

*See Paulsen *et al*. [18]. NA = not analysed.

**Figure 1:**
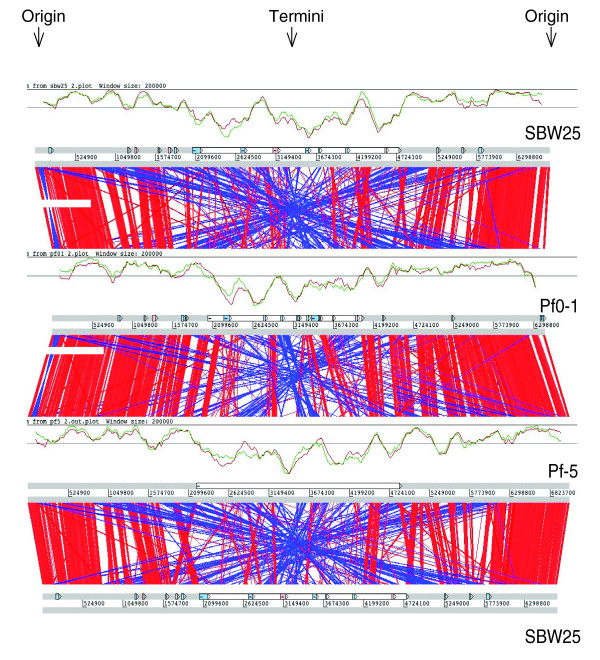
Comparison of amino acid matches between the complete six-frame translations of the whole genome sequences of *P. fluorescens *Pf0-1, SBW25 and Pf-5 genomes. The analysis was carried out using Artemis Comparison Tool and computed using TBLASTX. Forward and reverse strands of DNA are shown for each genome (dark grey lines). The red bars between the DNA lines represent individual TBLASTX matches, with inverted matches colored blue. Graphs show the density of CDSs with orthologues in the other two *P. fluorescens *strains (red and green lines). Window size is shown on the graphs. The thin grey lines show the genome average orthologue density. The white boxes on the DNA lines represent the variable regions around the termini as defined by these graphs (SBW25, 2.7 Mb; Pf0-1, 2 Mb; and Pf-5, 2.65 Mb). Blue and pink boxes represent the position of atypical regions and prophage, respectively.

Alignments of the whole genome sequences of *P. fluorescens *strains SBW25, Pf0-1, and Pf-5 revealed that the only long-range synteny among these genomes is confined to the origin of replication, with a gradual deterioration in both synteny and sequence conservation towards the replication terminus (Figure 1). There is also evidence of extensive reciprocal recombination around the terminus of replication, as commonly seen in other bacterial genomes [24] (Figure 1). Neither bacterium contains an accessory element (note that plasmid pQBR103 for which the complete sequence was recently reported [25] was acquired by SBW25 during a field release experiment [26], but this plasmid is not present in the originally isolated strain).

### Intra- and inter-species variation among *Pseudomonas genomes*

**Figure 2:**
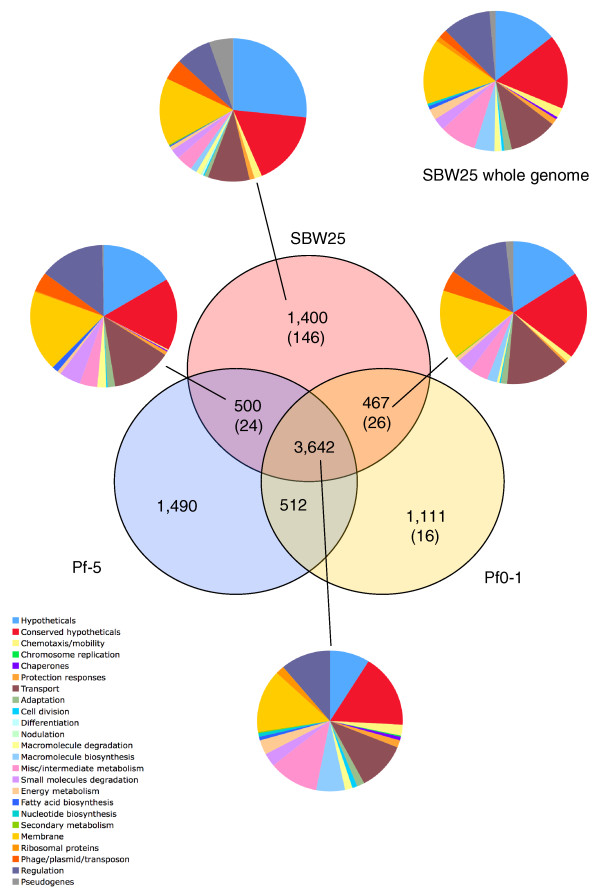
Venn diagram comparing the gene complements of *P. fluorescens *strains SBW25, Pf0-1 and Pf-5. The numbers of unique and shared CDSs are presented. Numbers in parenthesis are insertion sequence elements and pseudogenes. Pie charts indicate the absolute numbers divided into functional categories (see legend) for the complete gene complement of SBW25, the CDSs in common with the other two strains plus the core gene complement for all three.

Reciprocal FASTA analysis was used to identify orthologous gene sets shared among the three genomes. The distribution of genes and orthologues among the three *P. fluorescens *strains is non-random, with strain-unique genes being more common towards the replication terminus (Figure 1). This organization is similar to the accessory loci near the end of the arms (termini) of the linear chromosome in *Streptomyces coelicolor *A3(2), which are highly variable in both length and composition [27]. Of the total coding capacity, genes conserved among all three *P. fluorescens *isolates comprise 3,642 CDSs, representing 59.3%, 60.6% and 63.4% of the coding capacity in Pf-5, SBW25 and Pf0-1, respectively (Figure 2). A large proportion of the *P. fluorescens *genes (from 1,111 to 1,490 CDSs (22% to 27% of total coding capacity)) are found in just one genome (Figure 2). This finding contrasts with *Pseudomonas aeruginosa*, where the five sequenced isolates share a conserved core of 5,021 genes with only 1.4% (strain C3719) to 8.2% (strain PA2192) of genes unique to any one isolate [23]. It is possible that the overall low level of variation among the sequenced *P. aeruginosa *isolates reflects a bias created by restricting sampling solely to clinical isolates. If true, then it may be that the highly variable genomes of *P. fluorescens *are more representative of the true diversity of the *Pseudomonas *genus.

**Figure 3:**
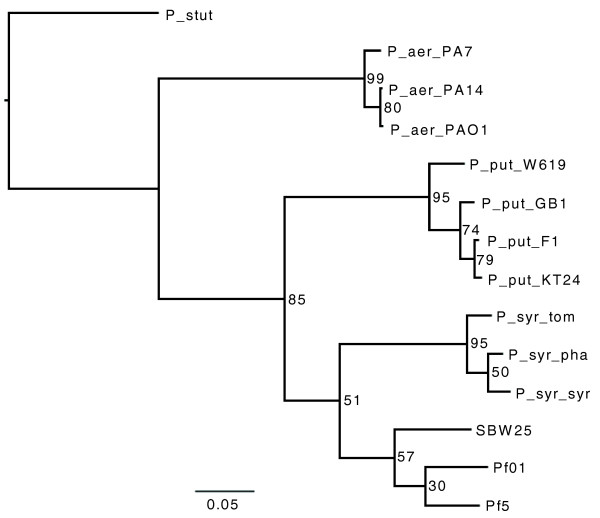
Phylogenetic tree of 14 different *Pseudomonas *species, based on 1,705 conserved genes: *Pseudomonas fluorescens *strains SBW25 (SBW25), Pf0-1 (Pf01) and Pf-5 (Pf5); *Pseudomonas aeruginosa *strains PAO1 (P_aer_PAO1), PA14 (P_aer_PA14) and PA7 (P_aer_PA7); *Pseudomonas syringae *pv. *syringae *B728a (P_syr_syr), pv. *tomato *DC3000 (P_syr_tom) and pv. *phaseolicola *1448A (P_syr_pha); *Pseudomonas putida *strains GB1 (P_put_GB1), F1 (P_put_F1), W619 (P_put_W619) and KT2240 (P_put_KT24); and *Pseudomonas stutzeri *strain A1501 (P_stut). Numbers on nodes represent percentages of individual trees containing that relationship. The scale bar corresponds to the number of substitutions per site.

When the reciprocal FASTA analysis was extended to include 11 other sequenced *Pseudomonas *species the conserved gene complement of these 14 *Pseudomonas *genomes was just 1,705 CDSs. This pseudomonad core gene-set falls below that previously estimated for the gamma-proteobacteria as a whole (2,049 CDSs [28]), underscoring the highly variable nature of this genus. This is also highlighted in Figure 3, which shows a majority rule consensus tree from the results of individual maximum likelihood analyses of the 1,705 core CDS amino acid datasets. The data strongly support the classification of *P. aeruginosa*, *P. putida*, and *P. syringae *isolates into species groups, with at least 95% of the single gene trees supporting the species distinction. In contrast, support for the classification of the three *P. fluorescens *isolates as a single species was relatively weak, supported by only 57% of single gene trees. Support for the intra-group relationships are not strong for any of the species examined and most likely reflects recombination among strains of each species [29]. Indeed, evidence of recombination in a number of different *Pseudomonas *species, including *P. aeruginosa *[30], and *P. fluorescens *[31] has been reported.

**Figure 4:**
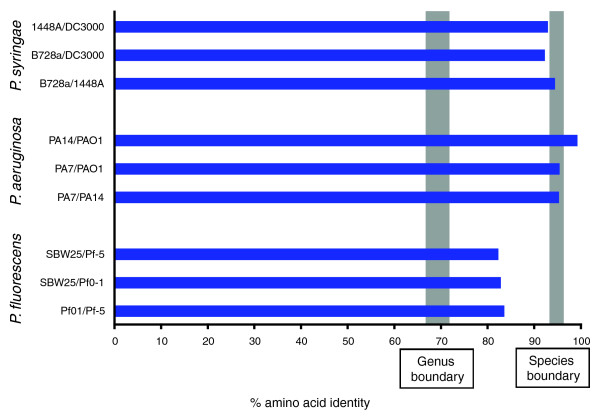
Average amino acid identities between pairs of *P. syringae*, *P. aeruginosa*, and *P. fluorescens *strains. The strain designations for the *P. fluorescens *and *P. aeruginosa *isolates and pathovar designations for the *P. syringae *isolates are as described for Figure 3. Genus and species boundaries are those used by Konstantinidis and Tiedje [32].

Average amino acid identities (AAIs) [32] were calculated using the pair-wise orthologous sets of CDSs from the three *P. fluorescens *strains as well as three *P. aeruginosa *strains and three *P. syringae *pathovars (Figure 4; Table 2). It is evident that the AAIs of the *P. fluorescens *strains are considerably lower than those found in *P. aeruginosa *and *P. syringae *and fall between the limits of genera and species as defined by Konstantinidis and Tiedje [32]. In addition, while unique sequences in each genome were excluded from AAI analyses, the relatively low number of orthologous sequences within the *P. fluorescens *genomes further calls the species grouping of these strains into question. However, we note that the AAI of orthologues located close to the replication origin ranges from 84.6% to 85.6%, whereas the AAI range for orthologues nearer the replication terminus is 75% to 77.5%: the genome wide AAI ranges from 82.2% to 83.4%. These regional differences require consideration before using AAI to infer relatedness.

**Table 2:** AAIs of the orthologous CDSs of *P. fluorescens *and *P. aeruginosa *strains and *P. syringae *pathovars

Strain pairing	AAI
*P. fluorescens*	
Pf0-1/Pf-5	82.786
SBW25/Pf0-1	81.784
SBW25/Pf-5	81.589
	
*P. aeruginosa*	
PA7/PA14	94.974
PA7/PAO1	95.070
PA14/PAO1	98.887
	
*P. syringae*	
pv. *syringae*/pv. *phaseolicola*	94.238
pv. *syringae*/pv. *tomato*	92.025
pv. *phaseolicola*/pv. *tomato*	92.597

AAI, amino acid identity.

Based on the genomic criteria provided by Goris *et al. *[33] for defining species, the three *P. fluorescens *strains could indeed be different species. In fact, our analysis is in agreement with previous studies that have shown - based on *gyrB *and *rpoD *nucleotide sequences - *P. fluorescens *to be a complex composed of two major lineages [34], with Pf-5 and Pf0-1 belonging in the *P. chlororaphis *and SBW25 in the *P. fluorescens *lineage. Similar conclusions have come from DNA-DNA hybridization and average nucleotide identity scores [33] and the genome signature (genome-specific relative frequencies of dinucleotides) [35]. Given the small sample of genomes, it seems premature to redefine the species '*P. fluorescens*' at this time. It should also be noted that our analysis shows the three *P. fluorescens *strains to group more closely to each other than to any other member of the *Pseudomonas *genus (Figure 3; Table 2).

### Functional analysis of the SBW25 gene complement

Analysis of the conserved genes present in the three *P*. *fluorescens *strains provides results that are typical of other soil dwelling bacteria [36,37]. For example, SBW25 and Pf0-1 carry an abundance of regulatory genes (>300 each), and genes encoding motility and chemotaxis-related functions (>100 each) as well as genes specifying membrane and transport functions (>1,000 each).

Also typical for pseudomonads, the genomes of SBW25, Pf0-1 and Pf-5 lack 6-phosphofructokinase, required for conversion of β-D-fructose 6-phosphate to β-D-fructose 1,6-bisphosphate (although the gene for 1-phosphofructokinase is present) and these strains are therefore unlikely to carry out glycolysis. Nonetheless, each genome possesses genes predicted to specify the enzymes phosphogluconate dehydratase and 2-keto-3-deoxygluconate 6-phophate aldolase, which are necessary for utilization of glucose via the phosphorylative Entner-Doudoroff pathway.

**Figure 5:**
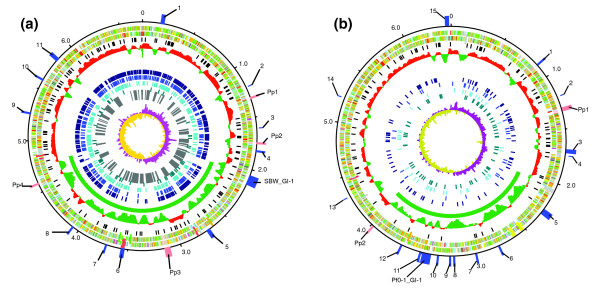
Circular genome maps of *P. fluorescens *strains SBW25 and Pf0-1. **(a) ***P. fluorescens *SBW25. From the outside in, the outer most circle shows atypical regions (blue boxes) and prophage-like regions (pink boxes) numbered according to Supplementary Table 3 in Additional data file 3; circle 2, scale line (in Mbps); circles 3 and 4 show the position of CDSs transcribed in a clockwise and anticlockwise direction, respectively (for color codes, see below); circle 5, location of IVET EIL fusions (black); circle 6, graph showing density of CDSs with orthologues (red) and those unique to SBW25 (green) compared to *P. fluorescens *Pf0-1 (window size 50,000 bp, step size 200); circle 7, *P. fluorescens *SBW25 variable region (green line); circle 8, IR1_g inverted repeats (dark blue); circle 9, R0 family of intergenic repeats (navy blue); circle 10, R2 family of intergenic repeats (light blue); circle 11, R5, R30, R178 and R200 families of intergenic repeats (aqua); circle 12, repeat deserts (ReDs; grey boxes); circle 13, GC skew (window 10,000 bp). CDSs were color-coded according to the function of their gene products: dark green, membrane or surface structures; yellow, central or intermediary metabolism; cyan, degradation of macromolecules; red, information transfer/cell division; cerise, degradation of small molecules; pale blue, regulators; salmon pink, pathogenicity or adaptation; black, energy metabolism; orange, conserved hypothetical; pale green, unknown; and brown, pseudogenes. Note that IR1_g repeats were not included in the ReD analysis because, based on their structure, we could not exclude the possibility that many of them simply represent transcription termination sequences. Where some ReDs appear to contain R-family repeats (for example, ReDs at about 6.1 Mb) there is actually more than one ReD, separated by a very small DNA region, that cannot be resolved in the figure. **(b) ***P. fluorescens *Pf0-1. From the outside in, outer most circle shows atypical regions (blue boxes) and prophage-like regions (pink boxes) numbered according to Supplementary Table 4 in Additional data file 3; circle 2, scale line (in Mbps); circles 3 and 4 show the position of CDSs transcribed in a clockwise and anticlockwise direction, respectively (for color codes, see above); circle 5, orthologues of SBW25 EIL - those EIL that are antisense in SBW25 are indicated by orthologues to the predicted CDSs on the sense strand; circle 6, graph showing density of CDSs with orthologues (red) and those unique to Pf0-1 (green) compared to *P. fluorescens *SBW25 (window size 50,000 bp, step size 200); circle 7, *P. fluorescens *Pf0-1 variable region (green line); circle 8, IR1_g inverted repeats (dark blue); circle 9, R5 family of intergenic repeats (navy blue); circle 10, R6 family of intergenic repeats (light blue); circle 11, R0, R1, R6-partial, R26, R30, R69, and R178 families of intergenic repeats (aqua); circle 12, GC skew (window 10,000 bp).

The extreme diversity evident in these three *P. fluorescens *isolates - both in gene content and sequence conservation - made a full metabolic reconstruction impractical in the context of *P. fluorescens *as a species. Such a reconstruction requires a greater number of complete genome sequences and an improved understanding of the nature of the *P. fluorescens *species. Instead, we focused on the direct identification of genes associated with colonization and survival in the plant environment using an IVET promoter-trapping strategy. This approach is the first step in a functional test of the prediction that the gene classes commonly associated with soil bacteria (outlined above) are determinants of their ecological performance. Previous attempts have exploited the IVET promoter-trapping strategy to identify genes up-regulated in the plant rhizosphere and soil environments [5-7]. While providing insight into a set of functionally significant genes, these studies have been based on the screening of partial genomic libraries and, therefore, the full spectrum of plant-soil-induced genes has not been identified. In order to obtain a comprehensive set of genes specifically active in the plant-soil environment, a full genome survey of plant- and rhizosphere-induced genes (collectively referred to as environment inducible loci (EIL)) in SBW25 was undertaken using the IVET strategy developed by Gal *et al. *[6]. This strategy selects EIL on the basis of their ability to drive the expression of a promoterless copy of the reporter gene *dapB *('*dapB*) - a gene required for the biosynthesis of diaminopimelate (DAP), which is an essential component of the peptidoglycan layer of the bacterial cell wall. Active EIL fusions to '*dapB *allow growth by complementing a *dapB *deletion in the SBW25 host strain used for these experiments. The distribution of EIL in SBW25 is shown in Figure 5a, and putative Pf0-1 orthologues are shown in Figure 5b. EIL classified by function, and putative orthologues in Pf0-1 and Pf-5, are given in Supplementary Table 1 in Additional data file 1.

EIL were identified by screening a library consisting of 33,000 clones (62 independent ligation reactions) and analyzed in pools of 250 on *Beta vulgaris *(sugar beet) seedlings. Given a genome of 6.7 Mbp, a random library of 3 to 5 kb fragments, and assuming 3,000 promoters in the SBW25 genome, then the chance of a promoter not being included in this study is less than 0.01 (based on the Poisson distribution).

The plant-inducibility of the EIL-'*dapB *fusion strains recovered by IVET selection was verified for each of the 125 IVET fusion strains by their inability to grow on M9 (glucose) minimal medium in the absence of DAP (thus demonstrating that the fusions are transcriptionally silent *in vitro*). The ability of each fusion strain to colonize both the rhizosphere and the phyllosphere of non-sterile sugar beet seedlings was then re-checked (strains colonizing these environments contain fusions to genes that are transcriptionally activated in the plant environment) [6,11]. SBW25Δ*dapB *and an IVET negative-control strain, PBR393 [38], were used as controls and no colony forming units of either strain were recovered from either the rhizosphere or phyllosphere. Every putative SBW25Δ*dapB *strain carrying an EIL-'*dapB *fusion grew in the rhizosphere (the size of the initial inoculum more than tripled in the rhizosphere over the course of 3 weeks); 90 of these IVET fusion strains were also able to grow in the phyllosphere (cells recovered from the phyllosphere underwent at least 3 doublings in 3 weeks). Growth of all EIL-fusion strains was significantly impaired in M9 (glucose) minimal medium. These tests verify that the EIL fusions are expressed by SBW25 on plant surfaces, and that the EIL promoters are dependent on the plant environment for expression. Further studies to determine the precise function of individual EIL in the plant environment are on going.

The 125 genes shown to be specifically up-regulated *in planta *represent all major classes of genes found in SBW25: Pf0-1 and Pf-5 each have orthologues of 83 of the 125 IVET-identified genes. Of these, 73 genes are common to all three *P. fluorescens *strains (Supplementary Table 1 in Additional data file 1). These data confirm the importance of previously recognized activities [6], and those predicted from genome sequence analysis, including nutrient acquisition and scavenging, cell envelope function, metabolism, stress response, and detoxification. Interestingly, when compared with the results of a previously conducted (small scale) study using the DAP-based IVET strategy [6], only 4 of the 25 EIL recovered in that study were identified here. These included the cellulose biosynthetic locus *wss *(recovered on six independent occasions in this study), *fliF*, *glcA *and *fadE *(Supplementary Table 1 in Additional data file 1). The reasons for the relatively low overlap between the two studies is unclear, but perhaps reflects subtle differences in conditions for plant propagation, although the differences are more likely to reflect the particularly stringent criteria applied in this study in order for a putative plant-induced locus to qualify as an EIL. Nonetheless, of importance is the fact that genes of the same functional classes were obtained in both screens.

No validated '*dapB *IVET fusions were obtained for genes within the Rsp type III secretion system, which was previously identified by a different (pantothenate-based) IVET selection strategy [5]. Its low level of expression in the rhizosphere [11] is likely to be insufficient to restore competitive growth in the DAP-based promoter trapping strategy used in this study.

Regulators form a large class of EIL: the 17 predicted regulatory components include a sigma factor, LysR-type regulators, two component sensing systems, a di-guanylate cyclase, and a phosphodiesterase. Also included in this collection is an operon defined by EIL037 (*PFLU1114*-*1111*) whose four CDSs show remarkable complexity: a compound GGDEF/EAL/CheY protein (*PFLU1114*), followed by a predicted cytochrome C551 peroxidase precursor (*PFLU1113*), followed by two compound histidine kinases (*PFLU1112 *and *PFLU1111*). That this and other regulatory loci are actively transcribed outside of the laboratory environment supports the generally held assumption that the abundance of regulatory genes in *Pseudomonas *is important for life in complex environments [39].

Another notable '*dapB *IVET fusion is EIL082, which falls within a previously unrecognized non-ribosomal peptide synthetase (NRPS) biosynthetic gene cluster (*PFLU3215 *to *PFLU3228*) present in the non-core region of SBW25. The non-ribosomal peptide produced by this enzyme complex is specific to SBW25, because no orthologues of these NRPS genes exist in Pf0-1 or Pf-5. Interestingly, Pf0-1 also possesses a novel NRPS gene cluster (*Pfl01_2265-2267*) not present in SBW25 or Pf-5. There are three NRPS biosynthetic clusters in SBW25, four in Pf0-1 and three in Pf-5, including the pyoverdine biosynthesis cluster. The similarity shared amongst these clusters is limited to conservation of the functional domains, with no full length identities. There is little conservation of the order of the functional domains. The production of pyoverdine is one of the defining characteristics for *P. fluorescens *and yet the genome has shown great diversity both in the pyoverdine clusters and in the other non-ribosomal peptides that are made by *P. fluorescens*.

Genes with no significant matches to DNA or protein sequences in public databases comprise approximately 10% of the IVET fusions. On the basis of *in silico *analyses, the majority of these genes are predicted to encode membrane-associated proteins, suggesting their interaction with the external environment through uptake, export, or signaling.

A large class of EIL are fusions of non-predicted genes, oriented in the opposite direction to transcription of predicted CDSs (40 in total; see class XI, Supplementary Table 1 in Additional data file 1). 'Antisense' fusions of this type have been described previously [5,40]. It is possible that some of these fusions highlight *in silico *gene prediction errors; however, careful examination of these fusions renders this unlikely. Moreover, in a previous study both the sense CDS and antisense IVET fusion were found to encode proteins; furthermore, the IVET-identified 'antisense' gene was shown to be important for efficient colonization of soil [41]. We refrain from further speculation as to the significance of these 'antisense' fusions, but such a substantial number suggests there is much yet to learn about the potential role of these genes in the function of bacteria in their natural environments.

Despite evidence for a highly variable accessory region towards the terminus of replication, the distribution of EIL in SBW25 appears to have little or no bias toward any particular genomic location (Figure 5a, b). The 31 genes defined by EIL055 to EIL096 are within the variable region (see below) of the SBW25 genome while the remainder are within the core region. This even distribution indicates that many of the mechanisms favoring success in natural environments are conserved, while individual strains appear to possess accessory traits that are likely to confer niche-specificity.

### Repeat families

Whole genome alignments of SBW25, Pf0-1, and Pf-5 showed evidence for extensive within-genome recombination. In many bacteria this is driven by recombination between repeat sequences. However, in none of the *P. fluorescens *genome rearrangements were the recombined sequences flanked by rRNAs, tRNAs or known insertion sequence elements. To identify repetitive DNA sequences that may explain this intragenomic recombination, an exhaustive search for such sequences in SBW25, Pf0-1, and Pf-5 was performed.

Analysis of SBW25 revealed the presence of 4,357 repeat sequences representing 11.91% of the genome. These repetitive sequences ranged in size from 24 to 357 bps and comprised 1,199 intergenic repeats, 922 inverted repeats (IR1_g), and 2,236 intragenic repeats. This type of repeat expansion has been seen in other systems, where it is associated with a relaxation of selection on the genome. This can be associated with a recent change in niche, and the resulting evolutionary bottleneck [42,43], or with reduced selection because of small effective population size and absence of recombination [44]. However, as discussed below, this is not thought to apply here. The intragenic repeat families represent coding sequences for conserved protein domains within over-represented protein families; 1,293 represented just 4 protein domain families (as defined by Pfam; see Materials and methods) - ABC transporter, AMP-binding enzyme, response regulator receiver domain and the GGDEF domain.

The *P. fluorescens *intergenic repeat elements comprised 12 families on the basis of sequence conservation (Supplementary Figure 1 in Additional data file 2). An analysis of their distribution and frequency (Table 3) within and between genomes shows examples of both strain-specific and species-specific families. The repeat families R0 and R2 are represented more than 500 times in SBW25, but are either absent or rarely present in Pf0-1 or Pf-5. Conversely, repeat family R1 is abundant in Pf-5, but rarely present in Pf0-1 and absent from SBW25; repeat family R6 is present in Pf0-1 and absent from the genomes of the other two strains.

**Table 3:** Characteristics of the intergenic repeat elements present in *P. fluorescens *SBW25, Pf0-1, and Pf-5

Repeat family	Size (bp)	G+C%	SBW25	Pf0-1	Pf-5	Structure
R0	89	59.81	612	36	-	IRs: 24 bp
R1	80	68.31	-	9	621	IRs: 30 bp
R2	110	66.52	516	-	4	IRs: 43 bp
R5	50	65.07	2	52	-	
R6	177	59.09	-	50	-	IRs: 34 bp
R6-partial	120	59.61	-	9	-	-
R26	352	60.76	-	20	-	-
R30	120	58.47	4	15	-	-
R69	62	64.67	-	12	-	-
R87	58	68.94	-	-	106	-
R178	101	61.86	18	28	9	IRs: 11 bp (internal)
R200	320	60.25	47	-	8	-
Total elements	-	-	1,199	231	748	-
						
Inverted repeats*						
IR1_g	23-28	66.52		125		-
IR1_g	21-28	64.7	922			-
IR1_g	23-27	65.2			36	-

* IRg_1 elements vary slightly in size, but all match closely with the consensus sequence and structure.

### Structural organization of the *P. fluorescens *intergenic repeats

Detailed analysis of the repeat sequences revealed that five families possess a complex structure consisting of two identical inverted repeats (IRs) that flank a variable size core region (Table 3). The IRs generally show a higher average G+C content than the genome as a whole (64.7%; the genome average is 60.5%), while the G+C% content of the variable core region sequences is closer to the genome average. Structural predictions made with these repeat sequences show that they readily form hairpin secondary structures, with the IRs forming the stem and the variable core region forming the loop.

Three repeat families, R0, R2 and IR1_g, are of particular interest given their disproportionately high numbers in SBW25 relative to Pf0-1 and Pf-5 (Table 3). The IRs of R0 and R2 are identical to those found flanking two different insertion sequence elements unique to strain SBW25 at locations 50373465038275 (*PFLU4572A*) and 63871926388340 (*PFLU5832*), respectively. It is possible that the IRs of repeat families R0 and R2 are recognized by the two insertion sequence element-encoded transposases *in trans*, which might explain why the elements have become over-represented in the SBW25 genome. If this is true, then these repeats are likely to represent miniature inverted-repeat transposable elements (MITEs), only very few of which have been reported in bacteria [45].

In addition to the ability to form stem-loop structures, the IR1_g repeats also possess the consensus sequence for the repetitive extragenic palindromic repeats (REP) family, which were originally thought to be specific to *P. putida *KT2440. The functional significance of the *Pseudomonas *REPs awaits elucidation, but they may play a role in transcription termination or provide binding sites for the DNA gyrase [46].

Since many of these repeat families can form stem-loop structures, they have the potential to act as transcriptional terminators. We therefore examined the transcription orientation of the genes flanking repeat elements to look for bias. In describing this analysis we use 'Head' to refer to the 5' end of a CDS and 'Tail' to refer to the 3' end. Using this nomenclature there are four transcriptional orientation states (including CDSs on both the forward and reverse DNA strands) for the CDSs that lie on either side of a repeat element: Tail-repeat-Head (forward strand) (→ →), Tail-repeat-Tail (→ ←), Tail-repeat-Head (reverse strand) (← ←) and Head-repeat-Head (← →). We compared the frequency of each of the four states with all CDS pairs that lacked an intervening repeat element. The frequency of the four orientation states among CDS pairs that flank repeat elements was significantly different from that of CDS pairs that do not (SBW25, *P *< 0.0005; Pf0-1, *P *= 0.016; Pf-5, *P *< 0.0005). For those CDS pairs that do not flank repeat elements the Tail-Head (forward and reverse strand) orientation is predominant; for CDS pairs flanking repeats the most frequent orientation is the Tail-repeat-Tail (Supplementary Figure 2 in Additional data file 2). The Tail-repeat-Tail bias is prevalent for the largest three of the six intergenic repeat families present in SBW25 and for five of the nine repeat families in Pf0-1 (Supplementary Figure 3 in Additional data file 2). The selective pressure for the non-random distribution of repeats may derive from the predicted stem-loop (transcription terminator-like) structure; insertion of a repeat with a stem-loop structure between Tail-Head oriented CDSs within an operon would cause termination, thus disrupting these transcriptional units. The Tail-repeat-Tail biased distribution of these repeats probably reflects a 'least worst' location as insertion is less likely to cause aberrant transcription termination since termination of convergent transcription is likely to occur anyway. In addition, the Head-repeat-Head state, which could potentially disrupt promoters for one or both genes, occurs at a low frequency, particularly in SBW25 and Pf-5. These data would also suggest that the expansion of the intergenic repeats has been subject to selection. Consequently, it is unlikely that the repeat expansion seen in *P. fluorescens *results from the organism having been through an evolutionary bottleneck (this scenario is generally associated with random distribution of repetitive sequences) [43] and more likely that it is linked to a lack of selection against increased genome size.

### *P. fluorescens *repeat deserts

Evident from the genome analysis are large regions of the SBW25 genome that lack any complex repeat families (R-family repeats; Table 3). We refer to these as repeat deserts (ReDs; Figure 5a). The SBW25 genome harbors 60 ReDs, which range in size from an arbitrary lower limit of 15.8 kb up to 176 kb and encode a total of 2,475 CDSs (40% of the coding capacity), of which 93.7% are unique to SBW25 compared to Pf0-1 and Pf-5 (Supplementary Table 2 in Additional data file 3). Because of the density of repeats in SBW25, the identification of ReDs was straightforward. In contrast, the lower number of repeats in Pf0-1 and Pf-5 makes definition of similar regions more difficult.

Two, not mutually exclusive, explanations for the lack of repeats in these regions exist: first, the ReDs comprise mostly essential genes that normally experience high purifying selection [47,48]; and second, the ReDs might have been recently acquired from a donor lacking repeat sequences. Indeed, examples of the former include the rRNA clusters, the ribosomal proteins cluster, the *wss *cluster (*PFLU0300 *to *PFLU0309*), which directs production of an acetylated cellulose-like polymer involved in formation of a microbial mat [49,50], and cell division proteins (*PFLU0940 *to *PFLU0953*, amongst others).

Recently acquired ReDs that have different dinucleotide frequencies to the above group contain CDS clusters that might confer niche specificity. One such example is the anthranilate synthase cluster (*PFLU1381 *to *PFLU1386*), which is unique to SBW25. Other examples found within ReDs include 'atypical' regions of the SBW25 genome, which show limited phylogenetic distribution, aberrant G+C% content or dinucleotide frequency compared with the genome average for *Pseudomonas *species (Supplementary Table 3 in Additional data file 3). These may reflect sequences acquired through recent gene transfer events [51]. While ReDs are not evident in Pf0-1, several such atypical regions have been identified (Supplementary Table 4 in Additional data file 3), and these are also free of repeats, as are all but one of the mobile genetic elements recently described in Pf-5 [52]. For example, SBW25 and Pf0-1 each carry multiple prophage-like elements, and both genomes have one probable integrative conjugative element (ICE)-like genomic island, *SBW_GI-1 *and the related island *Pf0-1_GI-1*, which have similarity to the genomic island *PFGI-2 *in Pf-5 [52]. *SBW_GI-1 *is located between partially duplicated tRNA_val _and is over 101 kb in length. Strengthening the possibility that this region is a hotspot for insertions, comparison of approximately 5 kb of unpublished sequences flanking the mupirocin biosynthetic cluster of *P. fluorescens *NCIMB10586 [53], which based on DNA sequence identity (generally 93% to 96%) and synteny is more closely related to SBW25 than Pf0-1 or Pf-5, indicates that the *mup *cluster is inserted adjacent to the same tRNA_val _tRNA_asp _tandem cluster as *SBW_GI-1*. *Pf0-1_GI-1 *defines a slightly smaller locus than *SBW_GI-1 *and lacks flanking insertion site duplications. These islands are related in structure to a family of ICEs, which include those found in other pseudomonads [54,55] as well as wider members of the gamma-proteobacteria such as *Yersinia *(*YAPI *[56,57]) and *Salmonella *(*SPI*-7 [58]). These elements are defined as having a conserved core carrying a type IV pilus operon and plasmid-related functions as well as a highly variable region, which carries genes involved in resistance and host adaptation. The reduction of the type IV pilus genes, and breakdown of the flanking regions in *Pf0-1_GI-1*, suggest these ICEs may be undergoing fixation in the genome, perhaps attributable to an important function of the cargo genes. The variable cargo regions of *SBW_GI-1 *and *Pf0-1_GI-1 *are summarized in Supplementary Tables 3 and 4 in Additional data file 3.

## Conclusions

*P. fluorescens *is an opportunistic species long recognized for its genetic, physiological and functional diversity [59]. The previously sequenced genome of isolate Pf-5 offered a glimpse of genome content and organization, but in the absence of comparative data sheds little insight into the extent of genomic diversity. The genome sequences of the two additional strains (SBW25 and Pf0-1) have provided the opportunity for comparative studies and show an unexpectedly high degree of among-genotype diversity. Typically, different isolates of the same species would be expected to show substantial overlap among core genes of the genome. For example, five sequenced genomes of *P. aeruginosa *share 80% to 90% of their gene content [23], whereas the three *P. fluorescens *genomes share just 61% of their genes, and have low average nucleotide identity [33] and AAI (this study), leading Goris *et al. *to suggest that these three isolates cannot be members of the same species. With further genome sequences, it will become possible to strengthen the species criteria using whole genome characteristics. The fact that these three strains group more closely to each other than to other members of the genus makes it tempting to describe *P. fluorescens *strains as members of a complex until more DNA sequence analyses provide a deeper understanding of the genetic structure of these populations.

The ecological significance of the genes specific to each strain also awaits further study, but the IVET-based analysis shows that at least some of the SBW25 genes are likely to be important in the plant environment. The fact that EIL fusions identify both core and accessory genes as ecologically relevant comes as little surprise given both the diverse range of core metabolic functions and the diversity of niches within which *P. fluorescens *exists. That a subset of the IVET-identified genes corresponds to orthologues in Pf0-1 and Pf-5 indicates conserved strategies for ecological success, and also the diversity of mechanisms employed.

The lack of synteny among the three strains marks a further defining feature of the species *P. fluorescens*. Previous studies of this species using restriction fragment length polymorphism showed a bewildering range of patterns - even amongst strains that were phenotypically indistinguishable [60]. The presence of numerous repeat sequences, particularly the intergenic MITE-like elements, provides a probable explanation. While the evolutionary origin of these elements is unclear, one likely consequence of the presence of numerous repeated sequences (between genes) is elevated levels of intragenic recombination. Although recombination between repeat sequences is to be expected, it seems that *P. fluorescens *can tolerate significant rearrangements without sacrificing performance. One striking example in SBW25 comes from the arrangement of genes involved in pyoverdine biosynthesis. In SBW25 these genes are distributed across seven different regions of the genome [17]; in Pf-5 and Pf0-1 (with fewer MITE-like elements) these genes are distributed across three [17] and five regions, respectively; in *P. aeruginosa *PAO1 (and other sequenced isolates) these are in two clusters separated by 11.5 kb; in *P. syringae *they reside within a single cluster [61].

Whole genome sequencing - particularly when combined with functional studies such as IVET - provides unprecedented insight into the functional activity of microbes. Despite their environmental significance, common saprophytic bacteria, such as *P. fluorescens*, have been the subject of relatively few genome-based projects. The addition of SBW25 and Pf0-1 to the list of genome-sequenced saprophytes is an important advance. It reveals the gene content of soil/plant saprophytes and shows that our prior appreciation of the diversity of the *Pseudomonas *pan genome was restricted. Since many isolates pathogenic to humans, animals, and plants are thought to have their origins in non-pathogenic environmental isolates, understanding the genomes of these saprophytes has implications for our ability to predict, monitor and understand the evolution of these pathogenic strains.

## Materials and methods

### Bacterial strains and sequencing

*P. fluorescens *strain SBW25 is an environmental isolate taken from the leaf surfaces of a sugar beet plant. A single colony of SBW25 was grown on LB agar and then grown overnight in LB broth with shaking at 28°C. Cells were collected and total DNA was extracted with a Gentra Puregene extraction kit (Qiagen, West Sussex, UK) according to the manufacturer's instructions. The DNA was fragmented by sonication, and several libraries were generated in plasmid vectors using size fractions ranging from 2 to 9 kb. The whole genome was sequenced to a depth of 9× coverage from 2 to 3 kb, 3 to 4 kb and 6 to 9 kb in pOTW12 and pMAQ1Sac_BstXI libraries using dye terminator chemistry on ABI3730 automated sequencers. End sequences from larger insert bacterial artificial chromosome (pBACehr 5 to 15 kb insert size) libraries were used as a scaffold. The sequence was assembled, finished and annotated as described previously [62], using the program Artemis [63] to collate data and facilitate annotation.

*P. fluorescens *strain Pf0-1 was isolated from bulk loam soil. It was grown overnight in LB broth with shaking at 30°C. Total DNA was extracted using a Wizard Genomic DNA Purification Kit (Promega, Madison, WI, USA). The genome of Pf0-1 was sequenced at the Joint Genome Institute using a combination of 3.7, 9.4, and 37 kb DNA libraries. Draft assemblies were based on 114,960 total sequence reads. All three libraries provided 5× coverage of the genome. A total of 470 additional reactions, 3 shatter libraries from PCR products, and 20 transposon bombs (*in vitro *transposon mutagenesis (EZ::TN<kan2>Insertion Kit; Epicentre, Madison, WI, USA) of plasmids to generate new primer sites for DNA sequencing) were necessary to close gaps and to raise the quality of the finished sequence. All general aspects of library construction, sequencing and gene prediction performed at the Joint Genome Institute were as previously described [64].

The sequences of SBW25 and Pf0-1 can be accessed using the accession numbers [EMBL:AM181176] and [GenBank:CP000094], respectively.

### Bioinformatic analyses

The genome sequences of *P. fluorescens *strains SBW25, Pf0-1 and Pf-5 were compared pairwise using TBLASTX analyses loaded on the Artemis Comparison Tool [65].

Orthologous CDSs in the three genomes were defined after comparing all-against-all running a reciprocal FASTA search of translated DNA with a 30% identity over 80% of the length of the CDSs as minimum similarity score. The results were used to calculate the average amino acid identities.

Pseudogenes were defined as CDSs that had one or more mutations that would ablate expression and/or lack start and/or stop codon; each of these possible inactivating mutations was subsequently checked against the original sequencing data.

Circular diagrams were plotted using DNAplotter [66].

#### Identification and analysis of orthologues in *Pseudomonas *genomes

Fourteen *Pseudomonas *species (*P. fluorescens *SBW25, Pf0-1, and Pf-5;*P. aeruginosa *PAO1, PA14 and PA7; *P. syringae *pv. *syringae *B728a, pv. *phaseolicola *1448A and pv. *tomato *DC3000; *P. putida *strains KT2440, W619, F1, and GB1; and *P. stutzeri *A1501) were compared all-against-all using a reciprocal FASTA approach (30% identity over 80% of the length as minimum similarity), yielding a set of 1,705 core genes shared between all these genomes. In a second step, the amino acid sequences of these core gene products were aligned (gene-wise) using MUSCLE version 3.52 [67] and poorly aligned regions were removed with Gblocks [68]. Maximum likelihood analysis of each alignment was carried out in RAxML version 7.0.0 [69] using the JTT+gamma model. A majority rule consensus of the 1,705 individual trees was built using the consense module of Phylip to assess the agreement between the individual trees.

### Identification and analysis of repetitive sequences in *P. fluorescens*

In order to analyze the repeat elements and their distribution in the genome of SBW25, we firstly concatenated three *P. fluorescens *genomic sequences (SBW25, Pf0-1 and Pf-5). Running the Repeatscout [70] algorithm on the concatenated sequence yielded 122 repeat families, of which 103 include intragenic repeats, mostly Pfam domains, and 19 intergenic repeat families. For each of the 122 families we built a multiple sequence alignment using CLUSTAL [71] and manually curated the alignments using JalView [72]. Using each of the multiple alignments obtained, we built a profile hidden Markov model (HMM) using the HMMER package version 1.8.4. The 122 HMMs were searched against the concatenated sequence (leading and lagging strand). HMMs can be trained on a dataset of sequences and can predict, in a probabilistic framework, more distant members of this sequence family. The results obtained were manually curated to infer the number of distinct repeat families. The consensus of the intergenic repeat families and their HMM logos are provided in Supplementary Figure 1 in Additional data file 2. The HMM logos where produced using the LogoMat-M application [73].

Intergenic repeat families were initially predicted using the default parameters of RepeatScout: minimum number of copies per repeat family, 20; minimum repeat length, 50 bp; low complexity repeats were filtered out prior to the repeat prediction. In a second step, the predicted repeats were manually curated and very similar repeat families were merged under the same family, where possible. A multiple sequence alignment for each repeat family was used to train HMMs specific for each family. Each query genome was searched against those HMMs, using the HMMER package. Once the repeat families were built, using the HMM-based approach, the structure of each family was determined with visual inspection of the multiple sequence alignment; in case of complex repeat structure, with IRs being part of a repeat family, new HMMs were built to model the IRs of each family (if applicable) and used to search the three query genomes.

#### Atypical regions

A computer-based search through the SBW25 and Pf0-1 genomes using the Alien Hunter program [74] resulted in identification of several regions within these genomes that were termed 'atypical' due to differences in nucleotide features such as G+C% and dinucleotide frequency. A manual curation of the results is shown in Figure 5, and Supplementary Tables 3 and 4 in Additional data file 3.

### *In vivo *expression technology

Identification of EIL from SBW25 was based on the IVET strategy as described previously [5,6]. Libraries were constructed in pIVETD by cloning partial Sau3AI digested genomic DNA. Libraries were maintained in *Escherichia coli *and moved into *P. fluorescens *SBW25Δ*dapB *by conjugation. Library screening took place on non-sterile sugar beet seedlings maintained in non-sterile vermiculite pots [5]. Fusions were recovered after 3 weeks of selection (rather than the 2 weeks used previously [6]) by plating homogenized plant material on selective plates. Integrated genomic fusions from strains recovered from the plant environment were mobilized into *E. coli *by conjugative cloning [75]. The identity of recovered fusions was determined by sequencing inserts from recovered plasmids (see [5,6] for details).

## Abbreviations

AAI: amino acid identity; CDS: coding sequence; DAP: diaminopimelate; EIL: environmentally induced loci; HMM: hidden Markov model; ICE: integrative conjugative element; IR: inverted repeat; IVET: *in vivo *expression technology; MITE: miniature inverted repeat transposable element; NRPS: non-ribosomal peptide synthetase; ReD: repeat desert.

## Authors' contributions

MWS, AMCT, RWJ, SBL, PBR, and NRT analyzed data and wrote the paper. AMCT and MWS manually annotated and curated SBW25 and Pf0-1 genomes. SRG, SDB, JP, GSV and SH analyzed data. ES and TSB were involved in sequencing, finishing, and quality control of the Pf0-1 genome sequence. KM participated in gene model annotation and curation. GLC and AMY analyzed the novel NRPS system. SRG, RWJ, GMP, XXZ, CDM, SMG, SACG, CGK, JGM, ZR, AJS, PBR, JH, ES, and CMT contributed to construction and screening of IVET libraries. DH, KS, LM, SR, RS, and MAQ were involved in sequencing, finishing, and quality control of the SBW25 genome sequence.

## Additional data files

The following additional data are available with the online version of this paper: Supplementary Table 1, listing environmentally induced loci in SBW25, and orthologues in Pf0-1 and Pf-5 (Additional data file 1); Supplementary Figures 1-3 (Additional data file 2); Supplementary Tables 2-4 (Additional data file 3).

## Supplementary Material

Additional data file 1 Supplementary Table 1: Environmentally induced loci in SBW25, and orthologues in Pf0-1 and Pf-5. Click here for file

Additional data file 2 Supplementary Figure 1 shows consensus sequences and HMM logos of *P. fluorescens *intergenic repeat families. Supplementary Figure 2 shows pie chart analyses of the CDS orientations in the three *P. fluorescens *strains. Left side, CDSs not flanking intergenic repeats; right side, CDS pairs flanking intergenic repeats. The orientation of the CDSs flanking intergenic repeats has a clear bias to the Tail-repeat-Tail (-> <-) orientation when compared to those CDSs that do not flank intergenic repeats. Supplementary Figure 3 shows the distribution of possible orientations of CDS pairs flanking each repeat sequence family, in **(a) **SBW25 and **(b) **Pf0-1. Click here for file

Additional data file 3 Supplementary Table 2 lists *P. fluorescens *SBW25 ReD coordinates and contents. Supplementary Table 3 lists atypical regions in *P. fluorescens *SBW25. Supplementary Table 4 lists atypical regions in *P. fluorescens *Pf0-1. Click here for file
