# Supplementary material for: Genomic and genetic analyses of diversity and plant interactions of Pseudomonas fluorescens
Source: Genome Biol. 2009 May 11;10(5):R51. doi: 10.1186/gb-2009-10-5-r51 (PMC2718517; doi:10.1186/gb-2009-10-5-r51)

Supplementary Figure 1

Repeat family	Consensus sequence
R0	nAAATGTGGGAGnGGGGCTTGCCCCCnGATnGCGGTGTGTCAGTCnnnAnnCATnnnnnnTGACTGAnnCAnnnnCCGCnAnTCGGGnGGCAAGCCCCnCTCCCACATTn
R1	nCnGTAGGAGCCGGCTTGCCGGCGAAGnnnnnnnnnnGGCCCnnnnnnnnnGnCnnGnGnCnnnnnnnnnnnnnnnnnnnnnGnnnnnnnnnnCTTCGCTGGCAAGCCAGCTCCTAC
R178	GnTTnATGGTGGCTgTGCGCAGGGCGCCTTCGGGnGCGCCGGnTnTnnnnntnnnnCCGGTCTACTAACCTGCGTAcAGCCGCCACCCnnTCnnGTTTAGTAGCGA
R2	TGTnGTGAGCGGGCTTGCCnnnnnnnnnnnnnnnnCCGCGCTnGGGCTGCGAAGCnGCCCCAAnAnnnnnnnnCnCCGCnnnTnTnTCAGnnAnAACnnGnGnnGnnnnGGTTTTGG<br>GGCnGnCTTCGCnnCCCAGCGCGGGGCAAGCCCGCTCACnACA
R26	CTTGGCGGCCTnnGGGCCGnCCAnGnTnTnnGGnnnGnTTTGGGTGnATATCCGTTnCTGCGGGTGCnGCnGCTGGCGGTTTCGCCCTTACGGCGAGTCACTTTTnnnnCA<br>AACGCCnnnnnnnnnnnnnnnnnnnAAAAGTAACCAAAAnnnGGCTTGnnCCCCnGCGTTCGGCCCnCTCGCTGnGGCTCGGGGTTCCTTCGCTCCGGGATTnATCCGGGGG<br>nCATCGCCTACGGTTTGCTTCGCTGCACCTCCTCTCGATGnnTnCGGCTnCGCCGnACGGTCGCTGCGCTCCCACCCCCGnnnnnnnnnnnnnnGATnAATCCCTCCACTCA<br>GCCTnCCGAnGnnGGGCCnGCnnnnnnnTCAAnAGCnGTACTCGAGCTAACGCTCATnGTGnTGAGTGGTGnnnnGnn
R200	nnnnnnnnnnnnnnnnnnnnnnnnnnnnnnnnnnnnnnnnnnnnnnnnnnnnnnnnnnnnnnnnnnnnnnnnnnnnnnnnnnncnnnGgGTACATATCCGTTATTTnGGTAACGGCnGCTaTGGGTTCCG<br>CnCTTACnGCGGGTCACTTTTGnnAAnnnnnAnnGCnnnnnnnnnnnnnnnCcAAAnGTAACCAAAAnnGCTCTTGCCCCAACACTCGGCACCTCGCnnAGGCTCGGTGTGCCC<br>nnAnTCCGnCATGnATnnGGGGGnnnCCGCCGCCAnCGCGCCATCCATGGCGCGnGGGCGGCTAAnCCGGCaTCCnTGCCGGnTTACCnCnCnnATCCnnGnCnnnnTnCGGCC<br>AGCGTGnTTTGACGGGGCGCCTnAGATCAAnAnCAAnAnCAnnnnnnAGAnCAnnAncnnnnnnnnnnnnnnnnnnnnnnnnnnnnnnnnnnnnnnnnnnnnnnnnnnnnnnnn
R30	CGGGCTACnTAAACCCGATCACTGATnnnTTCAGnGACCnnnnnAACnTTAnAnnCCGCCnCCnAGnCGCACAAGCCGGCGGATTCTGGCGTAnnnGTAGGCAAnGGCGCA<br>AGGnnnTGTAGCCn
R5	GGACGCGGAGCGTCCCnGGCTGCATTCCCACGCAGnAGCGTGGGAACGATCA
R6	CCCTCACCCTAGCCCTCTCCCnnnnnGAGGGnAGAGGGGACnGAnnnGGGGATnTTnGnGAnnTnCgCCGACCTGAnnnnnnnnnnnnnnnnnnGnnnnnnnnnnnnnnnnnnnnnnnnAAT<br>CCaTnnAATCGACnCGnTnTTTCAGGTCGATGTAnnnCGCAAngACnACCTCGGTCAGnnCCCTCTCCCTCCGGGAGnnnnnnnnnnnnnnAGGGCTnGGGnTGAGGG
R6-partial	nnnnnnnnnnnnnnnnnnnnnnnnnnnnnnnnnnnnGnnTTTnAAAACCTGAGTTCAnCTCGGTATTTCACGTCGGCGTAnCTCTCCCAAACAnCTCGGTCnGTCCCCTCTCCCTCCGG<br>GAGAGGGCTAGGGTGAGGGC
R69	nnGATCGTTCCCACGTCGAGGCGTCGAACCGTCTGCGTGGGAATGCAGnCCnnGACGCTCnGCGTC
R87	GGCnGGAGATCGCCAGGCAAGGCCCTGCGnnnnnnnnnnnnnnnGnCCTTTGCnGCAGCCTCGCnnnnGGCTCGGCAGCGGCTACAGG

R0

R1

R178

R2

R30

R5

R6

R69

R87

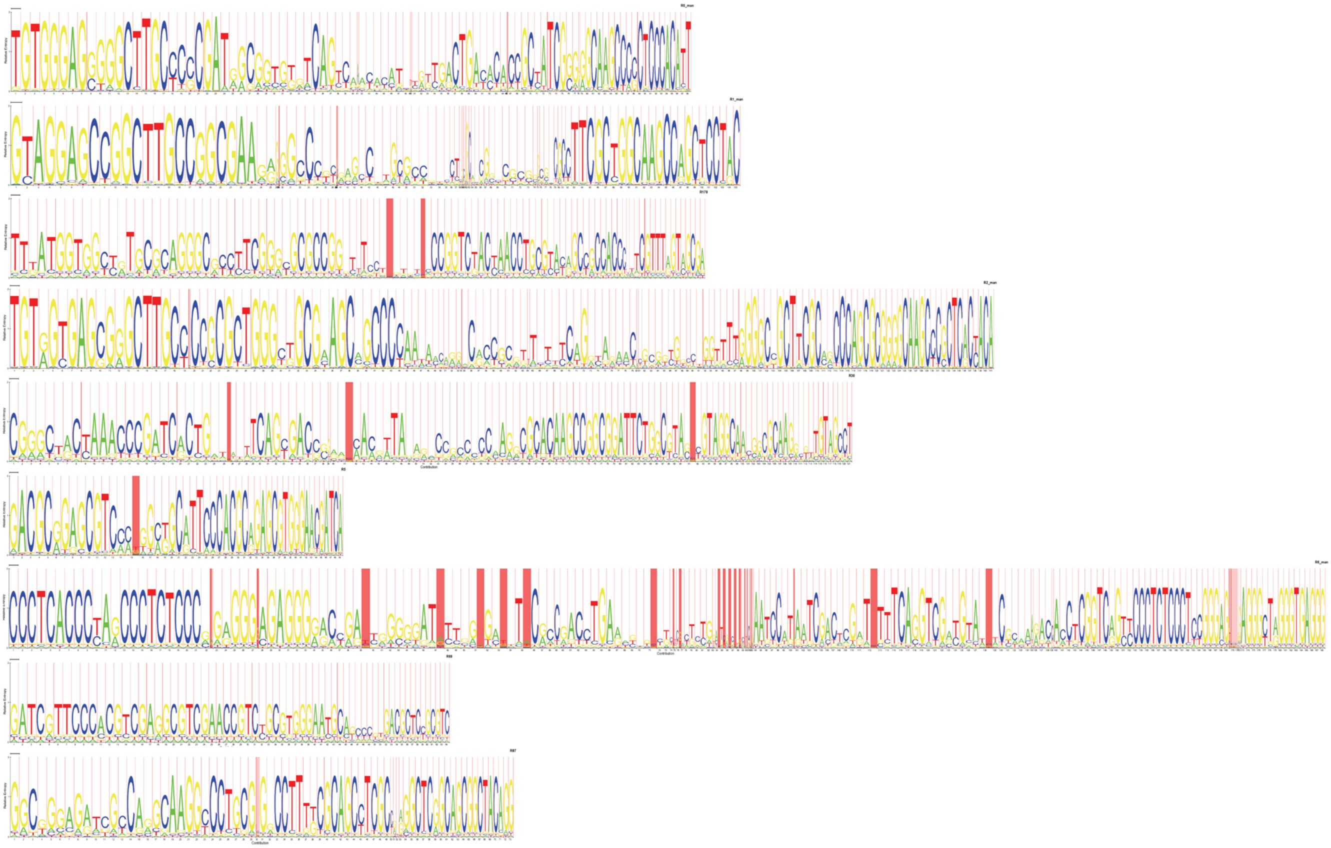

Supplementary Figure 2

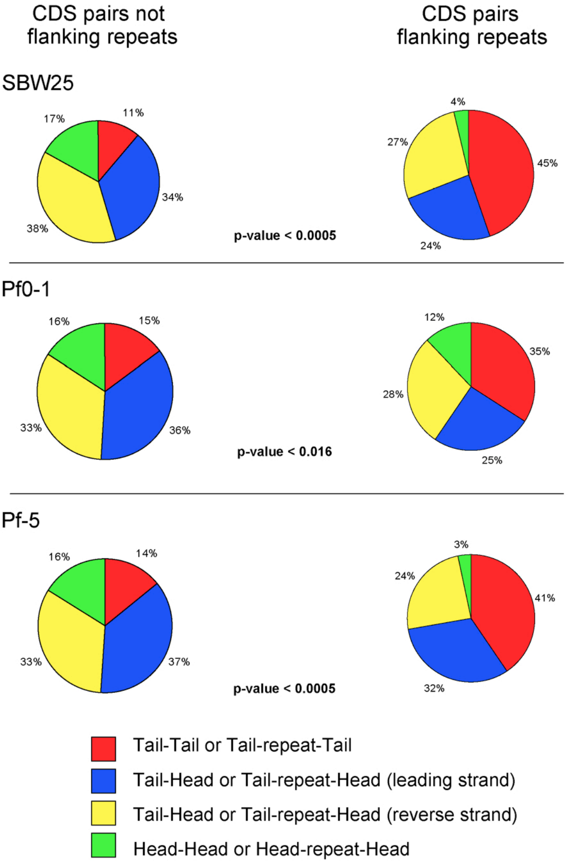

Supplementary Figure 3

a)

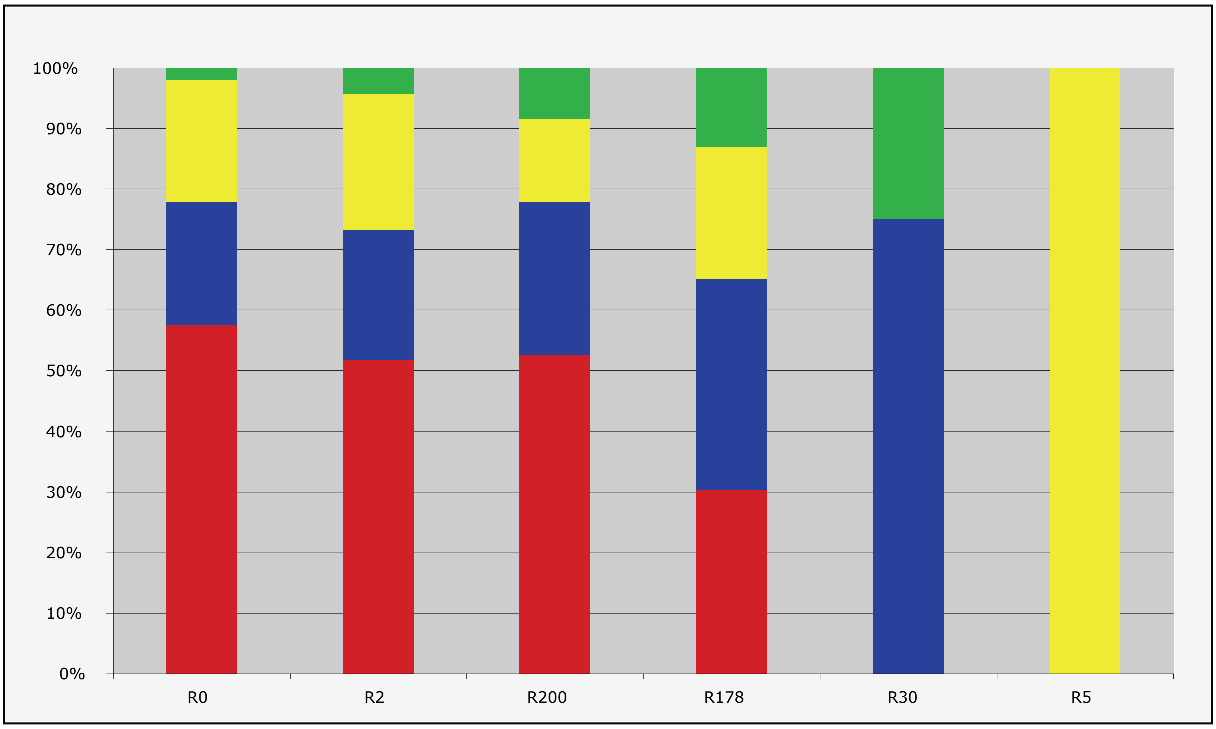

b)

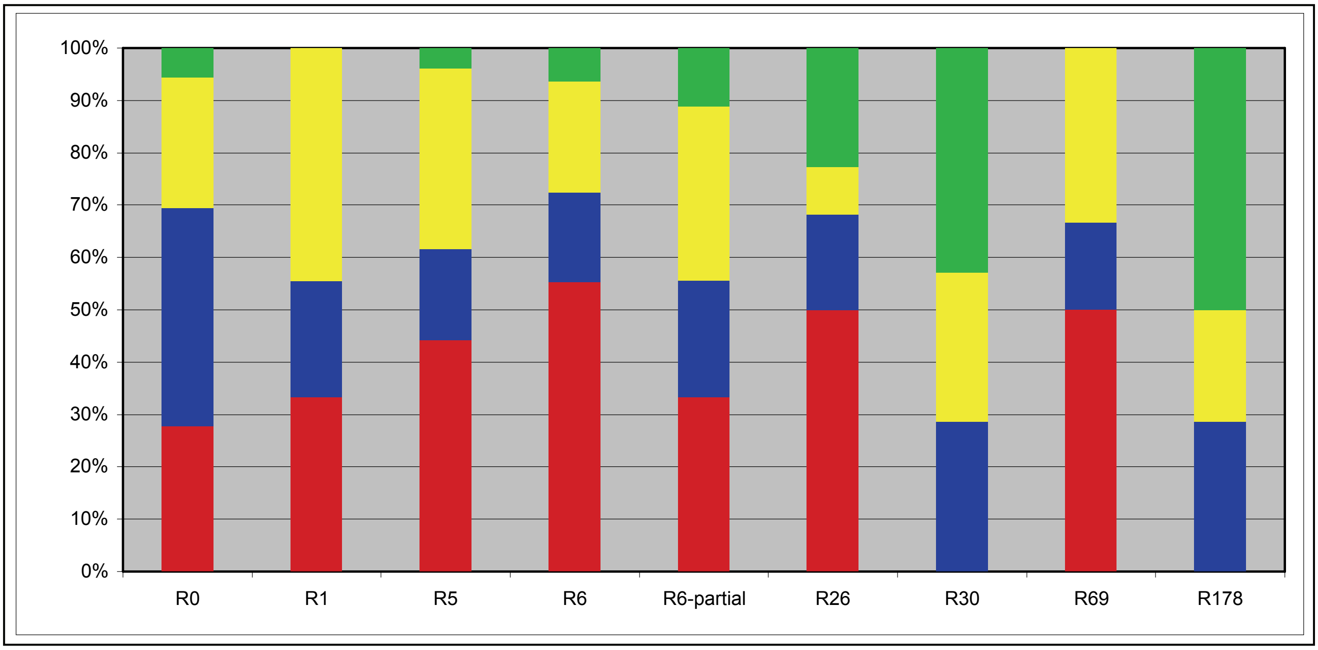

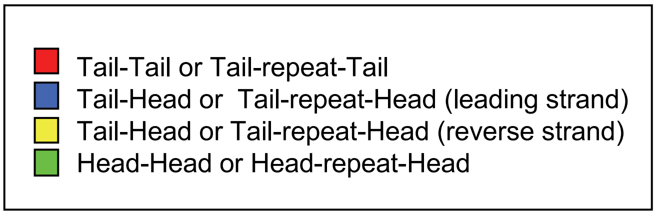

Supplement: Additional data file 2 — Supplementary Figure 1 shows consensus sequences and HMM logos of P. fluorescens intergenic repeat families. Supplementary Figure 2 shows pie chart analyses of the CDS orientations in the three P. fluorescens strains. Left side, CDSs not flanking intergenic repeats; right side, CDS pairs flanking intergenic repeats. The orientation of the CDSs flanking intergenic repeats has a clear bias to the Tail-repeat-Tail (-> <-) orientation when compared to those CDSs that do not flank intergenic repeats. Supplementary Figure 3 shows the distribution of possible orientations of CDS pairs flanking each repeat sequence family, in (a) SBW25 and (b) Pf0-1. [file gb-2009-10-5-r51-S2.pdf]
