# Supplementary material for: Genomic and genetic analyses of diversity and plant interactions of Pseudomonas fluorescens
Source: Genome Biol. 2009 May 11;10(5):R51. doi: 10.1186/gb-2009-10-5-r51 (PMC2718517; doi:10.1186/gb-2009-10-5-r51)
Supplement: Additional data file 3 — Supplementary Table 2 lists P. fluorescens SBW25 ReD coordinates and contents. Supplementary Table 3 lists atypical regions in P. fluorescens SBW25. Supplementary Table 4 lists atypical regions in P. fluorescens Pf0-1. [file gb-2009-10-5-r51-S3.pdf]

Supplementary Table 2 – *Pseudomonas fluorescens* SBW25 Repeat Deserts (ReDs) coordinates and contents.

Name	Coordinates	Repeat family flanking	Content	Mobile element: GI or prophage	IVET
ReD1	6700391..16700	R0 – R0	ATP biosynthesis cluster, chromosome partitioning proteins, iron-related RNA polymerase, tRNA modification protein, ribonuclease P, ribosomal protein. DnaA, DnaN, recF, gyrB, lipid A.	N	EIL148
ReD2	76775..103725	R0 – R2	PolA, cytochromes c4 and c5 precursors, alginate biosynthesis two-component system regulator, ABC transport system.	N	N
ReD3	1064107.139832	R0 – R0	NAD(P) transhydrogenase cluster, rRNAs.	N	N
ReD4	149271..185909	R0 – R0	ABC transport system, adhesin, hypotheticals.	Right hand side overlap with PFI-1	EIL003
ReD5	302209..349176	R2 – R0	Rhamnose modification-related cluster, *wss* cluster[13], amino acid transport system	N	EIL007<br>EIL008<br>EIL009<br>EIL010<br>EIL011<br>EIL012<br>EIL013<br>EIL014<br>EIL015
ReD6	448738..464568	R2 – R0	Fimbriae biosynthesis, chorismate biosynthesis.	N	N
ReD7	511813..530239	R2 – R2	Pyruvate dehydrogenase components	N	N
ReD8	530374..549712	R2 – R2	LPS biosynthesis, nodulation-related protein, lipid A.	N	N
ReD9	783944..810129	R0 – R2	Detoxification, ABC transport system, metal transport system, D-alanine--D-alanine ligase A.	N	EIL022<br>EIL023<br>EIL024<br>EIL025<br>EIL026
ReD10	810265..843145	R2 – R0	Type III secretion system, purine biosynthesis, RspL sigma factor, RspR, tRNA modification,peptide chain release factor 1, molybdpterin and peptidoglycan biosynthesis-related proteins	N	N

ReD11	925043..946242	R2 – R2	Dipeptide ABC transport system, two-component system, tRNA synthetase	N	N
ReD12	968666..1001372	R0 - R2	tRNA related, rod shape-determining cluster, ribonuclease G, DNA gyrase-related, TCA cycle (1 CDS), superoxide dismutase, PTS sytem, major RNA polymerase (rpoX, rpoN), ABC transport system, MurA	N	N
ReD13	1028081..1064465	R0 – R0	DnaA initiator-associating protein, bacterial ribonuclease P class A, cell division cluster (mur), phospholipids biosynthesis	N	N
ReD14	1193856..1255778	R0 – R0	Fimbriae biosynthesis cluster, hypotheticals, 50S ribosomal protein L31 type B-1, plasmid maintenance system killer protein, cytochrome C551 peroxidase precursor, two-component system	PFI-2	EIL037<br>EIL038
ReD15	1287654..1319761	R2 - R2	Ribonuclease T, dihydroorotase, endonuclease, tRNA synthetase, ferredoxin I, DNA mismatch repair protein, phage-related CDSs, recombinase A.	N	N
ReD16	1491674..1537061	R0 – R0	RNA polymerase associated protein, low-affinity inorganic phosphate transport membrane protein, cachetuate catabolism cluster, chaperone, acriflavine resistance cluster	N	EIL040
ReD17	1548570..1619900	R0 - R2	Dihydrodipicolinate synthase, glutathione transferase, methylated-DNA--protein-cysteine methyltransferase, ribosomal small subunit pseudouridine synthase A, uracil-DNA glycosylase, putative phage integrase, sigma factor + anti sigma factor, heat-shock adaptation serine protease	PFI-3	EIL041<br>EIL042<br>EIL043<br>EIL044
ReD18	1736160..1783142	R0 – R0	Two-component system, fimbria biosynthesis cluster, malate:quinone oxidoreductase (TCA cycle), acetylornithine aminotransferase (arginine biosynthesis), phage-related CDSs	N	N
ReD19	1797305..1841989	R2 – R2	3-demethylubiquinone-9 3-methyltransferase, DNA gyrase subunit A, amino acid biosynthesis-related, 30S ribosomal protein S1, integration host factor beta-subunit, polysaccharide biosynthesis cluster, potassium transport (*kdp*) cluster	PFI-4	N
ReD20	1842083..1841989	R2 – R2	Potassium transport, deoxyribonuclease, ABC transport system, transcription elongation factor, organic hydroperoxide resistance protein	N	EIL049<br>EIL050
ReD21	1896109..1922246	R200 – R2	Heat shock protein, transposase, glutathione peroxidase, two-component system regulatory proteins, ribosomal subunit pseudouridine synthase, molybdenum cofactor biosynthesis cluster	N	EIL051
ReD22	1992359..2026424	R0 – R0	Branched amino acid transport system, heat shock protein, fatty acid biosynthesis, two-component system regulatory proteins, pyrimidine biosynthesis	N	EIL053<br>EIL054
ReD23	2048889..2199797	R0 – R0	Inserted prophage, cytochromes C and D, glycolysis, transposase, IS element, DNA primase, amino acid biosynthesis, *nodD*, ABC transport system, DNA repair, DNA topoisomerase, cobalamin biosynthesis-related, biotin carboylase (fatty acid biosynthesis), amino acid transport system, chromosome partitioning	PFI-5	EIL055<br>EIL056

			soj		
ReD24	2221657..2264761	R0 – R200	Regulatory proteins, redox-sensitive transcriptional activator, amino acid biosynthesis, protein modification, rRNA, penicillin-binding protein, DNA polymerase II	N	N
ReD25	2265118..2296308	R200 – R0	Adenylyl-sulfate kinase, cytosine deaminase, conserved hypotheticals	N	EIL057
ReD26	2368925..2407348	R0 – R0	Amino acid biosynthesis, Excinuclease uvrABC system protein C, phospholipids biosynthesis, riboflavin biosynthesis deaminase, stationary-phase survival protein	N	EIL061
ReD27	2407442..2438735	R0 – R5	ABC transport system, homocysteine S-methyltransferase, peptide methionine sulfoxide reductase, nodulation-related aspartate aminotransferase, integrase (pseudogene), two-component system regulatory proteins,	N	N
ReD28	2438787..2489403	R5 – R2	Two-component system regulatory proteins, phage integrase (pseudogene), GABA permease, cytochrome C family protein, isoquinoline degradation cluster, ABC transport system, carbon compounds degradation cluster,		EIL062
ReD29	2682121..2786890	R0 – R0	nucleoside-specific transport system, sorbitol dehydrogenase, integrase, transposase, DNA methyltransferase, NAD synthetase-related, formamidase, Pyoverdine biosynthesis cluster, lot of repeats at the C-term.	PFI-6	EIL067
ReD30	2949454..2989870	R2 – R2	NAD kinase, cell division protein FtsH, aminopeptidase N, conserved hypotheticals, pentose pathway oxidative branch-related CDSs, ABC transport system, nitrilase	N	EIL071<br>EIL072<br>EIL073
ReD31	3039980..3070840	R2 - R0	Regulatory proteins, alpha-amylase, trehalose synthase, sugar branching enzyme, tryptophan biosynthesis protein, enoyl-CoAhydratase, NADH pyrophosphatase	N	N
ReD32	3106214..3282957	R2 – R2	Prophage, phospholipids biosynthesis, ABC transport system, regulatory proteins, ABC transport system, tRNA, Magnesium transport, arsenical pump, molybdenum ABC transport system, DNA methyl transferase, DNA repair, cytochrome C proteins, glucose metabloism-related, glutathione reductase, *tnpT/tnpS* pahge cointegrases, peroxide detoxification-related proteins, glycerol metabolism	Prophage 1	EIL075<br>EIL076<br>EIL077
ReD33	3321665..3396401	R200 – R2	Methyl-accepting chemotaxis 3x, hypotheticals, cation transport, amino acid biosynthesis, translation initiaion inhibitor, methyl viologen resistance, cytochrome b.	N	EIL078<br>EIL079
ReD34	3396496..3423578	R2 – R2	Methyl-accepting chemotaxis, protease, carbohydrate kinase, cytochrome b561, two-component regulatory system,	N	EIL080
ReD35	3451484..3626165	R2 – R200	Drug resistance, adhesin, glycolysis, fatty acid biosynthesis, chemotaxis, multidrug resistance cluster, NRPS biosynthesis cluster (6 CDS followed by ABC transport system), type II secretion cluster - gsp, adhesin, cytochrome P, aromatic	PFI-7	EIL081<br>EIL082

			hydrocarbons catabolism cluster		
ReD36	3638485..3677888	R2 – R2	Dehydrogenases and hydratases cluster, phosphate ABC transport system, hypotheticals	N	EIL083<br>EIL084<br>EIL085
ReD37	3720303..3768850	R2 – R2	Glycogen synthase, glycogen debranching protein, coserved hypotheticals, 2x methyl-accepting chemotaxis, amino acid permease, ABC transport system, dihydrodipicolinate synthase, methionine biosynthesis-related proteins	N	EIL086
ReD38	3789886..3820116	R2 – R200	Methyl-accepting chemotaxis, proline/betaine transporter, fosmidomycin resistance protein, two component system regulatory protein	N	N
ReD39	3906199..3947434	R0 – R0	Fatty acid biosynthesis cluster, ABC transport system, conserved hypotheticals, regulators, lysine biosynthesis related,	N	N
ReD40	4149439..4179299	R0 – R2	Regulatory proteins, $tRNA^{Ser}$, murein biosynthesis-related protein, lipopolysaccharide biosynthesis-related protein, biopolymer transport protein cluster, hybrid sensory histidine kinase in two-component regulatory system, D-lactate dehydrogenase, arsenate reductase	N	N
ReD41	4204940..4253670	R0 – R0	tRNA methyl-transferase, adenylosuccinate lyase, isocitrate lyase, anaerobic respiration *nuo* cluster, ABC transport system.	N	N
ReD42	4322761..4348953	R2 – R0	Dehydrogenase E1 component, peptidyl-prolyl cis-trans isomerase, DNA-binding protein HU-beta, Clp P protease cluster, trigger factor Tig, 4 tRNAs, folic acid biosynthesis-related protein, penicillin binding protein precursor, ABC transport system.	N	N
ReD43	4526427..4621746	R2 – R0	Iron siderophore related, amino acid ABC transport, two-component systems, acriflavine resistance cluster, chemotaxis, degenerated prophage 2 (15Kb), integration host factor, phenylalanyl-tRNA biosynthesis, initiation factor 3, major cold shock protein, amino acid biosynthesis, endonuclease, carbon storage regulator, peptidase, ABC transport, amino acid biosynthesis, 3 tRNAs, amid methionine biosynthesis-related, pophosphoribosyltransferase	Prophage 2	EIL093<br>EIL094
ReD44	4828679..4863533	R0 – R0	N-acetylmuramoyl-L-alanine amidase, two-component regulatory system, thiol:disulfide interchange protein cluster, non-ribosomal peptide synthetase, RNA polymerase sigma factor	N	EIL098
ReD45	5072219..5109377	R2 – R0	Dihydroorotate dehydrogenase, cell invasion protein, invasion protein regulator (pseudogene), InvG secretory protein, nitrate-binding protein, nitrate reductase regulator response regulatory protein, nitrate transporter, uroporphyrin-III c-methyltransferase, outer membrane porin A protein, RNA polymerase sigma factor, zinc transport protein, phosphoenolpyruvate synthase, alanyl-tRNA	N	EIL107<br>EIL108

			synthetase, 2-methylcitrate dehydratase, aconitate hydratase		
ReD46	5109475..5136980	R0 – R2	2-methylcitrate synthase, carboxyvinyl-carboxyphosphonate phosphorylmutase, para-aminobenzoate synthase component I, transposase, phosphoadenosine phosphosulfate reductase, regulatory protein, cobalt-nickel-resistance system transmembrane protein, tRNA, enoyl-CoA hydratase	N	N
ReD47	5174731..5210284	R2 – R0	DNA polymerase III, delta' subunit, thymidylate kinase, 4-amino-4-deoxychorismate lyase, fatty acid biosynthesis cluster, 50S ribosomal protein L32, ribosomal large subunit pseudouridine synthase C, ribonuclease E, citrate and C4-dicarboxylic acids transport protein, 2x two-component regulatory system, phosphopantetheinyl transferase, ribonucleoside-diphosphate reductase alpha chain, flagella biosynthesis cluster	N	N
ReD48	5247133..5275632	R0 – R0	Acetyl-coenzyme A synthetase, ribonucleoside-diphosphate reductase beta chain, IS element, 5-methylcytosine-specific restriction enzyme, pseudogenes, regulators, 2x methyl-accepting chemotaxis proteins, glycerophosphoryl diester phosphodiesterase, cation transporter	PFI-10	N
ReD49	5324243..5352843	R2 – R0	*malE*, array of membrane protein, chromosome initiator inhibitor homologue, *sodB* (superoxide dismutase), tRNA hydrolase, aroP (amino acid permease)	N	EIL111<br>EIL112<br>EIL113
ReD50	5408661..5434549	R2 – R2	Arginine biosynthesis bifunctional protein ArgJ, preprotein translocase ATPase secretion component SecA (General Secretory Pathway), methyl-accepting chemotaxis protein, formyltetrahydrofolate deformylase, exodeoxyribonuclease I SbcB, ABC transport system, pyruvate kinase, fumarate hydratase	N	N
ReD51	5457586..5484345	R0 – R2	ABC transport system, pH-inducible protein involved in stress response InaA, two-component regulatory system, chaperone Hsp60 peptide-dependent ATPase heat shock protein GroEL, 10 kDa chaperonin Cpn10, beta-lactamase induction signal transducer AmpG	N	EIL115
ReD52	5534811..5568055	R0 – R0	Maltoporin, trehalose-6-phosphate hydrolase, PTS system, sucrose-specific IIBC component, trehalose operon transcriptional repressor, GMP synthase cluster, exodeoxyribonuclease VII large subunit, 2-isopropylmalate synthase, GTP-binding protein EngA, histidyl-tRNA synthetase, 1-hydroxy-2-methyl-2-(E)-butenyl 4-diphosphate synthase (isoprenoid biosynthesis), nucleoside diphosphate kinase, adrenodoxin family ferredoxin, DnaK-homologue chaperone Hsc66, chaperone protein HscB, cysteine desulfurase	N	N
ReD53	5570877..5603730	R0 – R178	General secretory pathway proteins SecFD, queuine tRNA-ribosyltransferase, S-adenosylmethionine:tRNA ribosyltransferase-isomerase, tRNA, integrase,	PFI-11	EIL117

			hypothetical proteins, chemotaxis two-component regulatory system, chemotaxis cluster,		
ReD54	5705305..5844071	R0 – R2	*redDCB* cluster (exodeoyribonuclease), rRNA cluster, valine biosynthesis cluster, PrrF RNA, iron ABC transport system, regulator for maltose metabolism, DnaK suppressor, glutamyl tRNA synthetase, two-component regulatory system, pcnB poly(A) polymerase, folic acid biosynthesis-related, pantothenate biosynthesis-related, *acs* acetyl-CoA synthetase, polynucleotide phosphorylase/polyadenylase, tRNA pseudouridine synthase B, 30S ribosomal protein S15, ribosome-binding factor A, protein chain initiation factor 2, transcription termination factor, general secretory pathway protein SecG, triosephosphate isomerase, phosphoglucosamine mutase, dihydropteroate synthase, cell division proteins, transcription elongation factor, cleaves 3' nucleotide of paused mRNA, carbamoyl-phosphate synthase large and small chains, dihydrodipicolinate reductase, chaperone proteins DnaJ (HSP40), and DnkA (HSP70), protein GrpE (HSP-70 cofactor), DNA repair protein, ferric uptake regulation protein, SsrA-binding protein, pyruvate dehydrogenase complex repressor, glycolate permease, 2x phage integrase, DNA invertase, transposase for insertion element, resolvase, ParB, RepB, 3x transposase, 2x methyl-accepting chemotaxis protein	PFI-12	EIL122<br>EIL123<br>EIL124<br>EIL125<br>EIL126
ReD55	6019312..6086514	R0 – R0	tRNA hydrolase, trans-aconitase, *ssb* (helix destabilising protein), *uvrA* (DNA repair), bacterioferritin, *katA* (catalase), ribosomal proteins cluster, *rpoA* (RNA polymerase), *secY* (protein translocase), elongation factors, *rpoC, rpoB* (RNA polymerases), *secE*, rRNA cluster, tyrosyl t-RNA synthetase, arginine biosynthesis	N	EIL130
ReD56	6095903..6149266	R0 – R0	ABC transport system, two-component regulatory system, heat shock protein, exported organic solvent tolerance protein, chaperone, 4-hydroxythreonine-4-phosphate dehydrogenase, dimethyladenosine transferase, bis(5'-nucleosyl)-tetraphosphatase, symmetrical, thiosulfate sulfurtransferase GlpE, multifunctional Cca protein, folic acid biosynthesis, *gcp* (endopeptidase), *dnaG* (DNA primase), *rpoD* (RNA polymerase sigma factor), coenzyme PQQ biosynthesis cluster, RmsY RNA.	N	EIL132
ReD57	6446838..6469294	R0 – R2	glycerol-3-phosphate transporter, 3-octaprenyl-4-hydroxybenzoate carboxy-lyase, transcription termination factor Rho, thioredoxin, ABC transport system, exopolyphosphatase	N	N
ReD58	6521563..6552986	R0 – R0	Alanine racemase, catabolic, endoribonuclease, D-amino acid dehydrogenase small subunit, leucine-responsive regulatory protein, phospholipase D, aldehyde	N	EIL143

			dehydrogenase AldH, 50S ribosomal protein L33, 50S ribosomal protein L28, DNA repair protein RadC, coenzyme A biosynthesis bifunctional protein, deoxyuridine 5'-triphosphate nucleotidohydrolase, acetylglutamate kinase, orotate phosphoribosyltransferase PyrE, exodeoxyribonuclease III, ribonuclease PH, guanylate kinase, DNA-directed RNA polymerase omega chain, guanosine-3',5'-bis(diphosphate) 3'-pyrophosphohydrolase,		
ReD59	6553084..6588759	R0 – R0	Biopolymer ABC transport system, ATP-dependent DNA helicase, serine/threonine protein kinase, membrane proteins, array of conserved hypotheticals, *clpB*2 (chaperone)	N	N
ReD60	6624316..6647404	R0 – R0	aspartate ammonia-lyase, L-asparaginase, acetylpolyamine aminohydrolase, putrescine/polyamine-binding periplasmic protein precursor, 2x pseudogene, oxaloacetate decarboxylase alpha chain, biotin carboxylase,	N	EIL145<br>EIL146

Supplementary Table 3 – Atypical regions in *P. fluorescens* SBW25.

**Atypical region***	**Coordinates**	**Size (bp)**	**% GC**	**Content**
1	174091..203173	29,083	54.06	Signal peptidase (*lepB*)<br>Right-hand flanked by phage integrase
2	1208323..1217423	9,101	57.79	Fimbrial/pili biosynthesis
Pp1	1304498..1317448	12,951	60.46	Prophage1
3	1604701..1611162	6,462	53.71	Integrase
Pp2	1738434..1754039	15,606	51.96	Prophage2
4	1812790..1837706	24,937	46.95	Polysaccharide biosynthesis cluster
SBW_GI-1	2050500..2152291	101,792	57.37	Cytochromes C and D related proteins<br>Reverse transcriptase<br>Nodulation factor<br>Putative dihydrodipicolinate synthase<br>Putative ornithine cyclodeaminase<br>All three modules of an ICE element but not the type IV secretion system, which is degenerated: *pilL* is a pseudogene
5	2690543..2734621	44,079	51.88	Transposase<br>DNA methyltransferase
Pp3	3107845..3166503	58,659	57.42	Prophage3 cargo: Type I restriction enzyme
6	3527807..3568311	40,505	64.41	NRPS cluster
7	3696641..3708729	12,089	51.02	Methyl accepting chemotaxis protein
8	4050846..4077035	26,190	60.4	Putative capsular polysaccharide biosynthesis cluster
Pp4	4568906..4584205	15,300	51.85	Prophage 4 LPS-modification protein
9	5249167..5259420	10,726	52.43	Ribonucleoside-diphosphate reductase<br>Methylcytosine-specific restriction enzyme
10	5577181..5589497	12,317	50.1	Integrase
11	5798702..5833789	35,097	54.06	Integrase<br>Resolvase<br>Plasmid partitioning protein<br>Methyl-accepting chemotaxis protein

*** labels used to mark islands on Fig. 5A**

Supplementary Table 4 – Atypical regions in *P. fluorescens* Pf0-1.

**Atypical region***	**Coordinates**	**Size (bp)**	**% GC**	**Content**
1	862372..879205	16,834	51.22	Two probable phage integrase genes<br>Cluster Pfl01_738 to Pfl01_741 similar to *Streptomyces avermitilis* MA-4680 genes SAV2932 – SAV2929
2	1204474..1208081	3,608	47.33	BRO-like protein similar to phage antirepressors<br>RloA and RloB-like proteins (similar to those in *Campylobacter jejuni*), associated with abortive infection loci
Pp1	1313118..1345654	32,537	61.28	Pf0-1 phage_1
3	1688565..1715680	27,116	61.38	Glycosylation island Fla variable region #1
4	1739601..1747664	8,064	54.57	Fla variable region #2
5	2246556..2308948	62,393	62.94	General secretion proteins<br>Cytochrome related<br>Curli production<br>Putative phage DNA-binding and addiction module proteins<br>Polysaccharide production and export
6	2763561..2785012	21,452	53.43	Regulators
7	2987790..3010213	22,424	54.4	Ku domain protein<br>Protease inhibitor
8	3188064..3201801	13,738	52.15	Regulatory genes<br>Probable IS*30* transposase
9	3212045..3223054	11,010	54.85	Poly(3-hydroxyalkanoate) depolymerase<br>Zinc, gluconate, and amino acid transport<br>Mn-containing catalase<br>ISPsy6-like element and transposase
10	3327971..3343998	16,028	56.93	Putative halvibrin HvnB<br>Auto-transported pectin lyase<br>Drug efflux system (similar to AcrAB)
PfGI-1	3389342..3474979	85,638	55.36	Remnants of IS*Ppu20* and IS*Psy6*<br>Integrase pseudogene<br>Cytochrome C-type biogenesis<br>Benzoate and catechol metabolism (distinct from benzoate metabolism genes *Pfl01_2316*, *2320-2327* found elsewhere in Pf0-1 genome)<br>Quinohemoprotein amine dehydrogenase subunits<br>Plasmid pWWO-like genes<br>Phage-like genes<br>Lytic transglycosylase<br>*pilL* pseudogene<br>Antirestriction<br>DNA repair<br>Topoisomerase
11	3489458..3506828	17,371	52.74	Integrases<br>Plasmid stabilization (addiction)
12	3662049..3672127	10,079	47.89	ATPase similar to those in chromosome partitioning<br>N-acetylmuramoyl-L-alanine amidase inserted between *gspI* and *gspC* genes
Pp2	3962319..3986561	24,243	47.42	Pf0-1 phage_2
13	4316241..4324233	7,993	45.16	IS*Pfl1* (IS*110* family)<br>Transposase
14	5221907..5229492	7,586	45.64	Two HNH endonucleases<br>DNA methylase gene
15	6378949..6418164	39,216	54.14	Integrated in Tn7 Target site<br>Tn7 transposase genes ABCD, possibly duplicated, inverted copy of *tnsD*<br>Duplications of CDSs from elsewhere in Pf0-1

*** labels used to mark islands on Fig. 5B**
